# Natural Language Processing differentiates between Italian individuals with Nonfluent/Agrammatic from Logopenic variants of PPA

**DOI:** 10.1002/alz70857_105208

**Published:** 2025-12-25

**Authors:** Petronilla Battista, Simona Aresta, Paola Santacesaria, Allegra Benzini, Cinzia Palmirotta, Serena Tagliente, Rosa Capozzo, Alessandro Introna, Stefania Tagliente, Christian Lunetta, Christian Salvatore, Alberto Benussi

**Affiliations:** ^1^ Maugeri Clinical Inistitute IRCCS, Bari, Italy; ^2^ Global Brain Health Institute (GBHI) ‐ UCSF, San Francisco, CA, USA; ^3^ Istituti Clinici Scientifici Maugeri IRCCS, Bari, ‐, Italy; ^4^ University School for Advanced Studies IUSS Pavia, Italy, Pavia, Italy; ^5^ Clinical and Scientific Institutes Maugeri IRCCS, Bari, Italy; ^6^ Di Venere Hospital, Bari, Italy; ^7^ University of Bari Aldo Moro, Bari, Italy; ^8^ "F. Miulli" General Hospital, Acquaviva delle Fonti, Italy; ^9^ Istituti Clinici Scientifici Maugeri IRCCS, Milan, Italy; ^10^ DeepTrace Technologies S.r.l., Milan, Italy; ^11^ IUSS, Pavia, Italy; ^12^ university of trieste, Trieste, Italy

## Abstract

**Background:**

Differentiating between logopenic (lvPPA) and nonfluent/agrammatic (nfvPPA) variants of Primary Progressive Aphasia relies on expert evaluations of speech and language production. The dichotomy fluency/non‐fluency is not enough to support the clinical diagnosis as an effortful production of sentences (including morphosyntactic and phonological errors) can be present in both variants for different reasons. Connected speech analysis greatly supports the phenotypical classification of PPA, targeting abnormal language production.

**Method:**

In this cross‐sectional study, 19 Italian patients with PPA (nfvPPA=9; lvPPA=10) underwent an audio‐recorded picture description task from the SAND battery. The speech data were analyzed using computational methods with the Computerized Language ANalysis (CLAN) software, allowing the extraction of linguistic features. Using a Mann‐Whitney non‐parametric test corrected for false discovery rate (FDR), we analyzed 45 linguistic features belonging to four linguistic levels. A machine‐learning (ML) model was trained to classify nfvPPA vs lvPPA.

**Result:**

We identified ten features belonging to 4 linguistic levels differentiating nfvPPA from lvPPA. These included, at the phonetic and phonological level: silent pause ratio; at the lexico‐semantic level: noun, adverb, article, and determiner ratios; at the morphosyntactic level: total number of utterances and utterance error ratio; and at the pragmatic/discourse level: total words, total morphemes, and idea density. The ML model reached a sensitivity and specificity> 90%.

**Conclusion:**

We implemented natural language processing to perform a machine‐learning classification based on connected speech samples of Italian‐speaking subjects. This approach has been applied for the first time to Italian PPA and demonstrated that key linguistic markers can be identified and compared across the two variants.

